# Pharmacological Rationale for Targeting IL-17 in Asthma

**DOI:** 10.3389/falgy.2021.694514

**Published:** 2021-08-30

**Authors:** Siti Farah Rahmawati, Maurice te Velde, Huib A. M. Kerstjens, Alexander S. S. Dömling, Matthew Robert Groves, Reinoud Gosens

**Affiliations:** ^1^Department of Molecular Pharmacology, University of Groningen, Groningen, Netherlands; ^2^Department of Pharmacology and Clinical Pharmacy, Institut Teknologi Bandung, Bandung, Indonesia; ^3^Groningen Research Institute for Asthma and COPD (GRIAC), University Medical Centre Groningen (UMCG), Groningen, Netherlands; ^4^Department of Pulmonary Medicine, University of Groningen and University Medical Center Groningen (UMCG), Groningen, Netherlands; ^5^Department of Drug Design, University of Groningen, Groningen, Netherlands

**Keywords:** asthma, IL-17, pharmacology, inflammation, airway remodeling

## Abstract

Asthma is a respiratory disease that currently affects around 300 million people worldwide and is defined by coughing, shortness of breath, wheezing, mucus overproduction, chest tightness, and expiratory airflow limitation. Increased levels of interleukin 17 (IL-17) have been observed in sputum, nasal and bronchial biopsies, and serum of patients with asthma compared to healthy controls. Patients with higher levels of IL-17 have a more severe asthma phenotype. Biologics are available for T helper 2 (Th2)-high asthmatics, but the Th17-high subpopulation has a relatively low response to these treatments, rendering it a rather severe asthma phenotype to treat. Several experimental models suggest that targeting the IL-17 pathway may be beneficial in asthma. Moreover, as increased activation of the Th17/IL-17 axis is correlated with reduced inhaled corticosteroids (ICS) sensitivity, targeting the IL-17 pathway might reverse ICS unresponsiveness. In this review, we present and discuss the current knowledge on the role of IL-17 in asthma and its interaction with the Th2 pathway, focusing on the rationale for therapeutic targeting of the IL-17 pathway.

## Introduction

Asthma is a respiratory disease that currently affects around 300 million people worldwide and is defined by coughing, shortness of breath, wheezing, mucus overproduction, chest tightness, and expiratory airflow limitation (1). Asthma treatments are categorized as controller medications (anti-inflammatories alone or in combination with long-acting bronchodilators), reliever medications (bronchodilators), and add-on therapies for patients with severe asthma (1). Asthma that is uncontrolled with a high dose of inhaled corticosteroids (ICS) and second controller (long-acting inhaled β2 agonists, montelukast, and/or theophylline) and/or systemic CS for at least 6 months is defined as severe asthma (2). A comprehensive review of the current understanding of severe asthma and its treatment has been compiled by Israel and Reddel (3). The current add-on treatment options for severe asthma are long-acting muscarinic antagonist (LAMA), leukotriene modifier, low dose azithromycin, and low dose oral corticosteroids (OCS), and biological drugs such as anti-IgE, anti-IL-5/IL-5R, or anti-IL-4R for type 2 severe asthma (1, 3).

Asthma is characterized by airway hyperresponsiveness (AHR) associated with chronic airway inflammation (1). Several types of airway inflammation have been recognized in the pathobiology of asthma (4, 5). Type 2 inflammation is well-studied and characterized by its downstream (granulocyte-macrophage colony-stimulating factor (GM-CSF), IL-3, IL-4, IL-5, IL-9, and IL-13) and upstream (thymic stromal lymphopoietin (TSLP), IL-25 and IL-33) cytokines. This Th2-high endotype is linked to orchestration of the eosinophil biology and is considered as an important type of inflammation in a significant subpopulation of asthmatics (4,notch6). Other mechanisms include Th1 inflammation importantly mediated by the production of interferon-gamma (IFNγ) and Th17 inflammation characterized by IL-17A, IL-17E, IL-17F, and IL-22 cytokine production, which leads to neutrophil activation via IL-8 (6). Both Th2 and Th17 pathways can stimulate airway inflammation, tissue fibrosis, and AHR. However, the Th17-dependent inflammation is considered to be less sensitive to steroids, which constitute the primary anti-inflammatory treatment for asthma control (7, 8).

Increased levels of IL-17 have been observed in sputum, nasal and bronchial biopsies, and serum of patients with asthma compared to healthy controls (9–13). Moreover, expression is more pronounced in moderate-to-severe than in mild asthmatics (10, 13–16). In mild-to-moderate asthmatics, the level of IL-17A in the airway submucosal layer was significantly increased, whereas IL-17F was higher in both mild-to-moderate and severe asthma (13). Patients with higher levels of IL-17 are classified as a Th17-high inflammation in asthma. So far, there is no biologic treatment for Th17-high asthmatics, as opposed to the effective biologics for Th2 high. In fact, Th17-high subpopulation has a relatively low response to Th2 biologics, rendering this a relatively more severe asthma phenotype (10, 11, 14, 15, 17, 18).

Th17-high asthma is often referred to as neutrophilic asthma. During the onset of asthma, stimulation of the Th17/IL-17A axis leads to the release of neutrophil chemoattractants and their accumulation in the airways stimulates the development of neutrophil-based asthma with increasing severity (14, 19). All the above indicates that IL-17 exerts multiple effects in the progression of asthma that differ from the classical and more treatable Th2 types of the disease. As there are no anti-inflammatory treatment options available for Th17-high patients, there is an unmet clinical need for effective therapeutic strategies targeting Th17-driven asthma.

## Molecular Control of Interleukin-17 Signaling

The IL-17 cytokine family was first identified in 1995 and consists of six known members: IL-17A–IL-17F, with their corresponding five receptors: IL-17 receptor (IL-17R)A–IL-17RE, wherein IL-17A and IL-17F bind to the same receptor (IL-17RA) (20, 21). Interleukin −17 exerts a variety of effects, including the attraction of immune cells and activation of inflammatory signaling in airway structural cells (22, 23). Th17 is the primary immune cell responsible for the secretion of IL-17A/F and its regulatory cytokines, IL-21, IL-22, transforming growth factor β (TGF-β), and tumor necrosis factor-α (TNF-α) (24). This distinct cell type constitutes a subset of cluster of differentiation 4 (CD4)^+^ T cells generated from Th0 cells by stimulation with low concentrations of TGF-β, IL-6, or IL-21 (25). Further, Th17 cells are maintained and matured by IL-23 (25, 26). When IL-23 binds to the IL-23 receptor (IL-23R) expressed by Th17 cells, it activates Janus kinase 2 (JAK2), which subsequently phosphorylates signal transducer and activator of transcription 3 (STAT3) that enters the cell nucleus to induce transcription of specific target genes (25). This signaling cascade triggers Th17 cells to express IL-17 (25, 27). Other types of immune cells that are known as the source of IL-17 include γδ T cells (induced by IL-23, IL-1, retinoic acid, β-glucan, or bacterial products), CD3^+^ invariant natural killer T (iNKT) cells (induced by IL-23 or glycolipid), lymphoid-tissue inducer (LTi)-like cells (induced by IL-23, IL-7, or bacterial product), natural killer (NK) cells (induced by IL-23, retinoic acid, or IL-15), and myeloid cells such as neutrophils (induced by IL-6 and IL-23) (28, 29).

The initially defined IL-17R, IL-17RA, is a type I transmembrane protein that consists of an extracellular domain, a transmembrane domain, and a cytoplasmic tail (20, 24). Interleukin-17RA serves as a co-receptor for IL-17A and IL-17F, the two best-known members of the IL-17 family (21). In humans, IL-17RA gene expression has been identified in B and T lymphocytes, epithelial cells, fibroblasts, smooth muscle cells, macrophages, bone marrow stromal cells, monocytes, and vascular endothelial cells (30, 31). This might explain the broad influence of IL-17 on the regulation of normal physiological as well as pathological responses (32).

**Figure 1:**
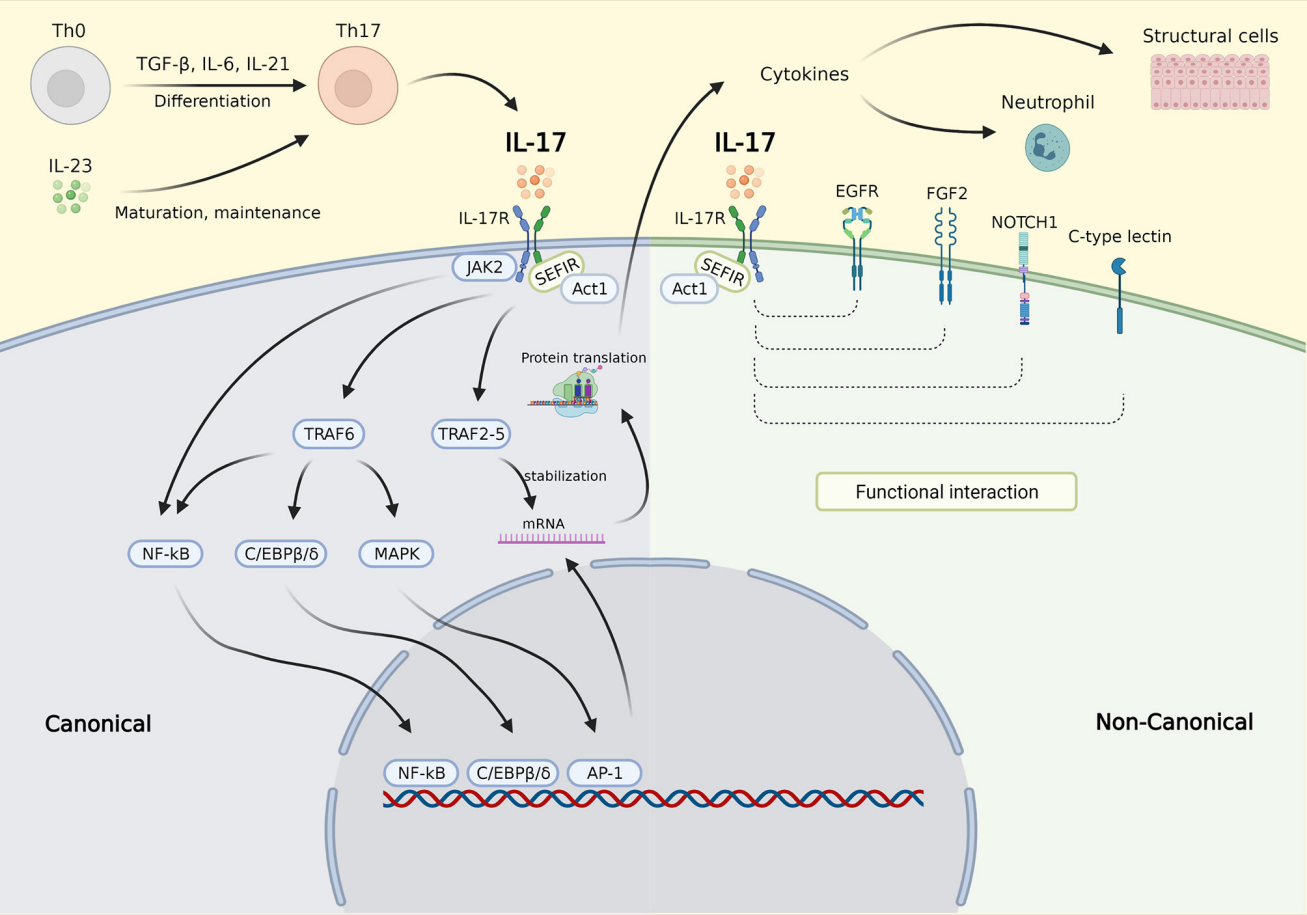
IL-17 signaling pathway. Th0 cells are differentiated into Th17 cells by stimulation with TGF-β, IL-6, and IL-21. Th17 cells are matured and maintained by IL-23. IL-23 also triggers Th17 to secrete IL-17. In the canonical pathway, upon binding to the IL-17R, IL-17 recruits Act1 to the receptor via interaction with SEFIR. Act1 further recruits several TRAFs needed for IL-17 transcriptional and posttranscriptional regulation. The inclusion of TRAF6 activates the NF-κB, C/EBPβ, C/EBPδ, and MAPK pathways, which then activate gene transcription (transcription phase). Activation of NF-κB was also carried out *via* JAK2 activation. Recruitment of the TRAF2-TRAF5 resulting in mRNA stabilization by stimulating mRNA stabilizing factors and/or by inhibiting mRNA destabilizing factors, thereby maintaining the translation of target genes (posttranscription phase). In the noncanonical pathway, the IL-17 complex functionally interacts with EGFR, FGF2, NOTCH1, and C-type lectin component resulting in enhanced molecular signaling in different cell types. The downstream effects of IL-17 signaling are mainly via the secretion of cytokines which then affect inflammatory cells such as neutrophil and structural cells, e.g. epithelial cell, smooth muscle cell, and fibroblast. IL-17, interleukin 17; IL-17R, IL-17 receptor; SEFIR, SEF/IL-17R; TRAFs, TNF receptor associated factors; NF-κB, nuclear factor kappa-light-chain-enhancer of activated B cells; C/EBPβ/δ, CCAAT/enhancer binding protein β/δ; JAK2, Janus kinase 2; MAPK, mitogen-activated protein kinase; mRNA, messenger ribonucleic acid; EGFR, epidermal growth factor; FGF2, fibroblast growth factor receptor 2; NOTCH1, NOTCH homolog 1. Created with BioRender.com.

The molecular control of IL-17 signaling is depicted in Figure 1. In the canonical pathway, upon binding to the IL-17R, IL-17 recruits Act1 to the receptor via interaction with SEF/IL-17R (SEFIR), a conserved region of the receptor in the cytoplasmic tail (32). Act1 further recruits several TNF receptor associated factors (TRAFs) needed for IL-17 transcriptional and posttranscriptional regulation (32). The mechanism involved in transcription is dependent on the inclusion of TRAF6 that activates the nuclear factor kappa-light-chain-enhancer of activated B cells (NF-κB), CCAAT/enhancer binding protein (C/EBP)β, C/EBPδ, and mitogen-activated protein kinase (MAPK) pathways. Recruitment of the TRAF2-TRAF5 complex occurs posttranscriptionally resulting in messenger ribonucleic acid (mRNA) stabilization by stimulating mRNA stabilizing factors and/or by inhibiting mRNA destabilizing factors, thereby maintaining the translation of IL-17 target genes (21, 32). The noncanonical IL-17 pathway was recently found to functionally interact with epidermal growth factor receptor (EGFR), fibroblast growth factor 2 (FGF2), NOTCH1, and C-type lectin receptor components (32). In addition, IL-17 is also known to act synergistically with other activators of NF-κB (TNF-α), STAT1 (IFN-γ), STAT6 (IL-13), and small mothers against decapentaplegics (SMADs) (TGF-β) (32).

The downstream effects of IL-17 signaling activation mainly occur via attracting immune cells, notably neutrophils (25, 30, 32). The IL-17/IL-17R signaling axis in lung epithelial cells induces the expression of granulocyte-colony stimulating factor (G-CSF) and neutrophil recruiting chemokines, such as C-X-C ligand 1 (CXCL1), CXCL2, and CXCL5 (33). These downstream effects include normal physiological responses, such as host defense regulation and tissue repair, as well as several pathological responses, including the development of several types of cancer (carcinoma, adenoma), aggravation of autoimmune diseases (psoriasis, rheumatoid arthritis, ankylosing spondylitis), asthma, and chronic obstructive pulmonary disease (COPD) (32).

## Th17 and Th2 Pathway Dynamics

As previously described, asthma consists of several types of airway inflammation (4, 6, 34). Furthermore, links between asthma phenotypes and inflammatory gene signatures have been reported (35). Sputum cells transcriptomic analysis from patients with moderate-to-severe asthma in UBIOPRED study revealed 3 transcriptome-associated clusters (TACs) (35). TAC1 was characterized by high gene signature enrichment associated with IL-13/Th2 type inflammation and type 2 innate lymphoid cells (ILC2), with low Th17, neutrophil activation, inflammasome, oxidative phosphorylation (OXPHOS), and aging signatures. On the other hand, TAC2 and TAC3 were described as Th2-low clusters, with high ILC1, neutrophil activation, and inflammasome signatures for TAC2, and high ILC3, Th17, OXPHOS and aging signatures for TAC3 respectively (35). Interestingly, eosinophilic inflammation correlates with TAC1 and TAC3, mixed granulocytic inflammation with TAC1 and TAC2, neutrophilic inflammation with TAC2, and a paucigranulocytic condition with TAC3 (35).

An IL-17A gene signature consisting of the 11 most upregulated genes in primary airway epithelial cells, including *CXCL3, CSF3, CXCL6*, vanin 1 (VNN1), solute carrier family 26 member 4 (SLC26A4), serum amyloid 1/2 (SAA1/2), melatonin receptor 1A (MTNR1A), TNFAIP3 interacting protein 3 (TNIP3), and *CXCL5*, has recently been identified (8). Although the presence of this gene signature has not yet been studied in asthma, this gene signature was further analyzed to evaluate the significance of its level of expression with regards to clinical characteristics of patients in two COPD studies, GLUCOLD and SPIROMICS (8). The IL-17 gene signature was found to be correlated with airway tissue neutrophils, airway tissue macrophages, and sputum neutrophils (8). Interestingly, it was negatively associated with change of forced expiratory volume in 1 s (FEV_1_) % predicted in all treatment arms and notably in patients receiving corticosteroids (CS), suggesting that the IL-17 gene signature not only represents a potential tool to assess airway disease severity but may also predict CS responsiveness (8).

The Th2 pathway in asthma can be recognized by increased secretion of IL-4, IL-5, and/or IL-13 resulting in eosinophilic inflammation (36). Moreover, the presence of an IL-13-inducible gene signature [i.e., chloride channel, calcium-activated, family member 1 (*CLCA1*), periostin (*POSTN*), and serine peptidase inhibitor, clade B (ovalbumin), member 2 (*SERPINB2*)] in airway epithelial brushings are considered as markers for this endotype (37). In addition to these specific gene signatures, asthma phenotypes can also be predicted by the expression of sputum six gene signature (6GS), which are *ALPL, CLC, CPA3, CXCR2, DNASE1L3*, and *IL1B* (38). The 6GS expression can significantly distinguish between eosinophilic asthma (high *CLC, CPA3*, and *DNASE1L3* expressions) vs. non-eosinophilic asthma, and between neutrophilic asthma (high *IL1B, CXCR2, ALPL* expressions) vs. non-neutrophilic asthma (38).

Although both Th2 and Th17 pathways are able to stimulate airway inflammation, tissue fibrosis, and AHR, only Th2 cell-mediated effects are CS sensitive (7, 39). In fact, treatment with CS further elevated the influx of neutrophils in the lung and upregulated IL-17-inducible chemokines, CXCL3 and CXCL1, in a preclinical model of allergen-induced asthma (39). Thus, patients with predominantly Th17-driven airway inflammation are likely less sensitive or even unresponsive to CS treatment.

Interestingly, the Th2 and Th17 pathways have been reported to regulate each other. Several studies showed that IL-13-induced IL-17 downregulation is mediated through the JAK/ STAT6-signaling pathway (7, 40–42). In a murine model of asthma, neutralization of the Th2 cytokines IL-4 and IL-13 increased Th17 cell number and neutrophilic inflammation in the lung (7). Accordingly, mice with IL-4R alpha deficiency exhibited elevated levels of IL-17 and decreased eosinophil recruitment into the airways (43). Furthermore, human Th17 cells express the IL-13Rα1 and stimulation with IL-13 prevented IL-17 secretion from these cells (41).

The influence of IL-17 on the IL-13 pathway is more complex. Interleukin−17A has been shown to moderately suppress several IL-13 inducible genes (*POSTN, CLCA1, SERPINB2*) in human bronchial epithelial cells (7). Moreover, asthmatics with high expression of the IL-17 gene signature (Th17-high) have low IL-13 gene signature expression and vice versa; patients with Th2-high asthmatic presented a depleted IL-17 gene signature, suggesting a reciprocal relationship between the pathways (7, 44). Furthermore, IL-17 decreased pulmonary eosinophil recruitment by downregulating the eosinophil chemokine eotaxin (CCL11) and thymus- and activation-regulated chemokine/CCL17 (TARC) as well as by reducing IL-5 and IL-13 production in murine lung (43). This implies that the effect of IL-17 is not limited to IL-13 expression and also affects other downstream cytokines in Th2 pathways.

Interestingly, simultaneous induction of IL-17A and IL-13 in a murine asthma model resulted in increased AHR compared to stimulation with IL-13 alone, whereas IL-17A alone had no effect (40). This indicates a synergistic interaction of these cytokines in AHR (40). Indeed, neutralization of both IL-17A and IL-13 inhibited eosinophilia and neutrophilia, mucus hyperplasia, and AHR in a preclinical model of allergen-induced asthma (7, 45). Therefore, targeting IL-17 and IL-13 pathways concurrently may result in a more effective treatment strategy.

In air liquid interface (ALI)-grown primary human epithelial cells, inhibition of heat shock protein 90 (HSP90) prevented goblet cell metaplasia stimulated by IL-13 or IL-17A (46). HSP90 is a cellular protein-folding factor involved in several physiological and pathological processes (47). The inhibitory effect was postulated to be due to interference with signaling driven by erythroblastic leukemia viral oncogene (ERBB) (ERBB1/EGFR and ERBB2-4), TGF-β, nuclear receptor coactivator 3 (NCOA3)/SRC3, and ets homologues factor (EHF), which are all relevant factors in IL-13 as well as IL-17-induced goblet cell metaplasia (46). In addition, Notch4 expression in the peripheral blood of murine asthma model stimulates regulatory T (Treg) cell destabilization and induces differentiation into Th2 and Th17 cells, via the Hippo pathway and Wnt axis, toward a Th17 and Th2 cell fate, respectively (48). Furthermore, Notch4 expression in Treg cells increases with asthma severity (48). These observations suggest that IL-13 and IL-17 signaling share upstream regulators, which would allow for the targeting of both inflammatory pathways via a shared regulator.

## The Relationship Between Interleukin-17 and Neutrophilic Asthma

Neutrophilic airway inflammation is defined as an inflammatory condition with sputum neutrophil cutoffs ranging from ≥60 to ≥76% (6). In asthma, this type of inflammation is related to reduced sensitivity to steroid treatment, lower forced vital capacity (FVC) % predicted, reduced FEV_1_ reversibility, and higher disease severity compared to patients with non-neutrophilic asthma (16, 49). The level of sputum neutrophils is one of the most discriminating factors in phenotyping patients with more severe asthma (50, 51). Although it is estimated that around 50% of severe asthma cases are neutrophilic, no therapeutic approach is available to specifically target the neutrophilic inflammation in asthma (6, 49).

The level of IL-17 in the airways is correlated with sputum (8, 11) and bronchial (15) neutrophil counts. The mechanism by which IL-17 regulates neutrophil activities has been increasingly studied over the past decade. It has been demonstrated that IFN-γ plus lipopolysaccharide (LPS)-stimulated neutrophils induce the production of CCL2 and CCL20, which bind to CCR2 and CCR6, respectively, on Th17 cells (52). These interactions resulted in Th17 activation that could be effectively inhibited by anti-CCL20 and anti-CCL2 antibodies (52). Moreover, in a mouse model of combined allergic asthma with *Haemophilus influenzae* infection, Th17 cell differentiation and neutrophil influx into the airway were significantly induced, resulting in allergic neutrophilic asthma features that were steroid resistant (53, 54). These data suggest that IL-17 might play an important role in neutrophilic asthma associated with airway infection. Indeed, neutrophilic asthmatics have a lower diversity of sputum microbiota compared with eosinophilic asthma, which correlates with airway infection in patients with asthma (55). Furthermore, patients with neutrophilic asthma have higher pathogenic bacteria, i.e *Haemophilus* and *Moraxella* taxa, and reduced common airway microorganisms, such as *Gemella, Porphyromonas*, and *Streptococcus* taxa, compared to eosinophilic asthma (55).

Conversely, activated Th17 cells affect neutrophils by promoting the production of CXCL8/ IL-8, a well-known chemoattractant of neutrophils, in the microenvironment and via GM-CSF, TNF-α, and IFN-γ release (52). This suggests reciprocal crosstalk between neutrophils and Th17 cells via these chemokines. However, neutrophils do not express IL-17RC; therefore, they cannot be activated directly by either IL-17A or IL-17F produced by Th17 cells (52). The effect of IL-17 on neutrophils is rather indirect by means of activating structural cells, including airway epithelial cells, fibroblasts, and airway smooth muscle (ASM) cells, to produce the above-mentioned cytokines and chemokines that in turn interact with neutrophils (33, 42, 43, 56–60).

## Disease Severity and Interleukin-17

The prevalence of severe asthma ranges from 3.6 to 6.1% in the total adult population-based asthma cohort, and is associated with an age over 50 years, nasal polyposis, impaired lung function, sensitization to mold, and female gender (51). Asthma that is uncontrolled with a high dose of ICS and second controller (long-acting inhaled β2 agonists, montelukast, and/or theophylline) and/or systemic CS for at least 6 months is defined as severe asthma (2). Uncontrolled asthma is clinically characterized by inadequate symptom control, frequent severe exacerbations, and airflow limitation despite bronchodilator use (2). Airflow obstruction in severe asthma might be due to structural alterations in the airway (airway remodeling) (3). Severe asthmatics have a higher risk of asthma exacerbation and morbidity (3).

The challenging task of targeting severe asthma is further complicated by the complex and diverse pathobiology of asthma. Phenotyping, which integrates biological and clinical characteristics, has been used to categorize asthma into several clusters (2); these represent the range of disease severity from mild-to-moderate asthma with predominantly eosinophilic inflammation to moderate-to-severe asthma with neutrophilic or mixed granulocytic inflammation (3, 50, 61).

Several studies have reported higher levels of IL-17, notably IL-17A and IL-17F, in sputum, nasal and bronchial biopsies, and blood of patients with severe asthma as compared to those with mild asthma (10, 11, 14, 15, 18). In fact, an IL-17 level of 20 pg/ml in serum was identified as an independent risk factor for severe asthma (14). Likewise, histological expression of IL-17F exceeding values of 23 cells/mm^2^ for bronchial and 19 cells/mm^2^ for nasal biopsies could be used to discriminate between mild and severe asthmatics (15). These findings suggest that IL-17 can be considered a biomarker for severe asthma.

The presence of IL-17 in asthma has been demonstrated in several preclinical and clinical studies. Interleukin-17-related cytokine expression was upregulated in nasal and bronchial biopsies of neutrophilic asthmatic patients just prior to an exacerbation, indicating a possible role of IL-17F in frequent exacerbators (15). This was in line with observations by Östling et al., who showed that IL-17-high asthmatics were at risk of frequent exacerbations (44).

More severe AHR is another hallmark feature of severe asthma. In a study that measured AHR by quantifying the response to methacholine in asthmatic children, AHR was reported to be positively correlated with serum IL-17A as well as the number of Th17 cells and the Th17/Treg ratio in peripheral blood mononuclear cells (PBMCs) (12). In a mixed Th2/Th17 mouse model of steroid-insensitive asthma, IL-17A was shown to be an independent contributor to AHR (62). In addition, the presence of Th17 cells in mice resulted in increased AHR that could not be resolved with dexamethasone treatment, whereas inhibition of IL-17A was effective (39, 40). These studies also showed that the effect of the IL-17 pathway on AHR were associated with the number of neutrophils, suggesting regulation by the IL-17-neutrophil axis (39, 40).

Furthermore, an association of IL-17 with lung function and steroid sensitivity is evident. Thus, the FEV1 value of asthmatics negatively correlated with the expression of IL-17F in bronchial and nasal biopsies as well as the number of Th17 cells in serum, serum IL-17A, and the Th17/Treg ratio (12, 15).

## Effects of Interleukin-17 on Airway Remodeling

Airway remodeling is an important feature of severe asthma and contributes to lung function reduction and obstruction of airflow (63). It is characterized by goblet cell hyperplasia, elevated submucosal extracellular matrix (ECM) deposition, thickening of reticular basement membrane (RBM), ASM cell hyperplasia and tissue hypertrophy, and changes in the bronchial microvasculature (64–66).

Changes in airway epithelium are considered hallmark features of airway remodeling. These changes include epithelial to mesenchymal transition (EMT), which is a phenotypic conversion of epithelial cells characterized by cell disaggregation as well as reduced epithelial (e.g., E-cadherin) and increased mesenchymal (e.g., vimentin, α-SMA) marker expression (67). Interleukin-17A has been shown to induce EMT of primary murine bronchial epithelial cells by inhibiting E-cadherin expression and stimulating vimentin expression via NF-κB activation (58). Interleukin−17A has also been shown to affect mucus production by airway epithelium. Exposure to IL-17A increased IL-13-stimulated expression of the goblet cell hyperplasia marker, chloride channel, calcium activated 3 (CLCA3), in mouse lungs via enhanced IL-13-induced STAT6 signaling (68). In line with these findings, a study using a mouse asthma model of Th17 inflammation showed a correlation between IL-17A expression and goblet cell number (69). Furthermore, IL-17A induced MUC5AC gene and protein expression through NF-κB activation in differentiated primary human bronchial epithelial cell cultured in ALI and a human bronchial epithelial cell line (HBE1) (70). These studies strongly suggest that IL-17A elevates mucus production. Another epithelial response to IL-17 is related to ECM production. Hyperreactivity of STAT3 in T-lymphocytes resulted in the expansion of Th17 cells in murine lung parenchyma and overexpression of matrix metalloproteinase-9 (MMP9) (71). Matrix metalloproteinase-9 simulates the degradation of ECM proteins, including collagen and laminin, which is consistent with airway epithelial remodeling.

Interleukin-17A also exerts its influence on fibroblast function. Primary mouse lung fibroblasts stimulated with IL-17A *in vitro* showed elevated TGF-β1 secretion and procollagen1a2 (proCol1a2) expression (69). The TGF-β1 secretion by fibroblasts was further increased after co-stimulation with IL17A and wingless-type mouse mammary tumor virus (MMTV) integration site family, member 5A (Wnt5a), suggesting the involvement of a IL-17A/Wnt5a/TGF-β1-axis mediating the effects of lung fibroblasts on airway remodeling (69). In agreement with these studies on murine fibroblasts, supernatant of IL-17A-exposed primary human parenchymal fibroblast culture increased collagen synthesis and TGF-β1 secretion in primary human lung fibroblasts (72). Moreover, fibroblast proliferation induced by these supernatants was attenuated by anti-TGF-β1, indicating that IL-17A-stimulated fibroblast activation was mediated by autocrine TGF-β1 expression (72).

In another study, normal human lung fibroblasts expressed IL-17RA and proliferated in response to 1 and 10 ng/ml IL-17A (73). Stimulation with IL-17A also increased α-SMA expression, indicating fibroblast-to-myofibroblast transdifferentiation. Moreover, when cultured on soft (polyacrylamide) gels, IL-17A-stimulated ECM deposition (collagen type I and fibronectin) in primary human lung fibroblasts (73). These responses were carried out via NF-κB signaling and inhibition of JAK2, but not JAK1/3, prevented these fibrogenic responses (73). Interestingly, IL-23, a known regulator of IL-17 expression, also employs JAK2 in its mechanism (25). Furthermore, IL-17A upregulated fibronectin and collagen-III protein expression in primary normal human parenchymal fibroblasts but not in normal human bronchial fibroblasts, indicating that IL-17A effects on ECM production are dependent on fibroblast phenotype (60).

Airway smooth muscle tissue hypertrophy, a result of cellular hyperplasia and/or hypertrophy, represents a prominent feature of airway remodeling (64). In one study, a mouse model of mixed Th2/Th17 asthma was developed by intranasally transferring allergen-pulse LPS with adenosine 5'-triphospate (ATP)-activated dendritic cells. In this model, IL-17A production was correlated with α-SMA, a marker for mesenchymal cells and ASM thickness (69). The expression of Th17-related cytokines receptors, such as IL-17RA, IL-17RC and IL-22R1, has been detected in primary human ASM cells (74). The corresponding cytokines, IL-17A, IL-17F and IL-22, were shown to promote ASM cells migration in a dose-dependent manner, which could be inhibited by blockade of these receptors, implying receptor-dependent effects (74). Furthermore, the IL-17A and IL-17F responses could be partially prevented by a p38 MAPK inhibitor, whereas IL-22-stimulated effects were attenuated by NF-κB inhibition (74). Interleukin-17A, IL-17F, and IL-22 induce proliferation of primary human ASM cells as well (75). These effects were mediated by ERK 1/2 MAPK for IL-17A and IL-17F, and by both ERK 1/2 MAPK and NF-κB signaling for IL-22. These cytokines were also shown to decrease apoptosis and promote cell survival; in addition to effects on migration and proliferation, this could potentially contribute to ASM mass thickening (75).

## Interleukin-17 and Steroid Sensitivity in Asthma

Inhaled corticosteroids constitute the cornerstone controller therapy in the treatment of asthma of all severities (1). They exert their anti-inflammatory effect via transcriptional and posttranscriptional mechanisms (76). Inhaled corticosteroids transcriptional mechanisms interfere with pro-inflammatory and anti-inflammatory genes that are induced by inflammation. Upon binding to glucocorticoid receptors (GRs), and predominantly GRα, ICS form a heterodimer complex that translocates into the nucleus and binds to a DNA recognition site known as glucocorticoid response element (GRE) in the steroid-responsive-gene promoter regions (76, 77). This induces transcriptional coactivator molecules, such as cyclic AMP element binding protein (CREB), to acetylate core histones resulting in activation of anti-inflammatory gene transcription (76, 77). On the other hand, ICS suppress transcription of inflammatory genes via interaction with pro-inflammatory transcription factors, such as NF-κB and activator protein 1 (AP-1), which reverses histone acetylation and prevents pro-inflammatory gene transcription (76). Inhaled corticosteroids also inhibit the MAPK pathway via MKP-1 stimulation resulting in the blockade of the expression of several pro-inflammatory genes. Posttranscriptionally, ICS promote the degradation of pro-inflammatory mRNA that was previously stabilized by certain pro-inflammatory cytokines, thereby alleviating inflammation (76).

With a more advanced understanding of asthma pathophysiology, particularly with regards to ICS responsiveness, there is a growing body of research on reduced ICS sensitivity in asthma. Failure of ICS to adequately inhibit inflammatory molecular and/or clinical features can be caused by several mechanisms, including GR modification, increased GRβ expression, increased pro-inflammatory transcription factors (e.g., NF-κB and AP-1), immune mechanisms (e.g., elevated Th17 activity, decreased Treg activity), and defective histone acetylation and deacetylation (76, 78). Detailed reviews on the mechanisms of reduced ICS sensitivity in asthma are available (76, 78).

Interleukin-17 has been associated with reduced steroid sensitivity in inflammatory diseases, including asthma. An *in vitro* study showed that Th17 cells differentiated from naïve CD4^+^ T cells from antigen-specific TCR-transgenic mice are unresponsive to dexamethasone and continue to produce IL-17A and IL-22 despite successful translocation of GRα into the nucleus (39). Transfer of these Th17 cells into mice challenged with ovalbumin was sufficient to induce increased CXC chemokine secretion, neutrophil infiltration, and AHR, all of which were less responsive to dexamethasone treatment (39).

As indicated, Th17-high asthma is associated with neutrophilic airway inflammation. In a mouse model of acute exacerbation of chronic asthma, it was shown that although dexamethasone treatment suppressed airway inflammation associated with eosinophil and T-lymphocyte recruitment, it did not prevent the influx of neutrophils or the development of AHR, suggesting that Th17/ neutrophilic inflammation is more resistant to dexamethasone treatment (79). Furthermore, in this model, dexamethasone inhibited histone acetyl transferase (HAT) activity in the lungs but failed to reverse the increased NF-κB activity and reduction of histone deacetylase-2 (HDAC2), which is one of the mechanisms of reduced ICS sensitivity (79).

The potential mechanisms underlying the effects of IL-17 on reduced steroid sensitivity have also been evaluated *in vitro* using human cells. Irvin et al. found that Th2/Th17 cells in the bronchoalveolar lavage (BAL) from asthmatic patients, which presented with higher IL-17 levels in the BAL, were resistant to dexamethasone-induced cell death. In addition, the expression level of MAP-ERK kinase 1 (MEK1), an inducer of AP-1, was elevated, suggesting its involvement in reduced ICS sensitivity (80).

In human airway epithelial cells, the ability of budesonide to inhibit TNF-α-induced IL-8 production was significantly reduced by IL-17A pretreatment (81), which could likely be attributed to enhanced phosphoinositide-3-kinase (PI3K) pathway activity and a subsequent reduced HDAC2 activity in response to IL-17A (81). Moreover, primary airway epithelial cells from asthmatics have been shown to express significantly higher levels of GRβ after IL-17A/F stimulation (82). These studies suggest that IL-17 might directly contribute to reduced ICS sensitivity of the airway epithelium.

Reduced ICS sensitivity has also been linked with the high expression of GRβ. GRβ is an alternatively spliced form of GRs, which binds to glucocorticoid receptor element (GRE) in the DNA but not with CS (76, 83). Thus, it competes with GRα and acts as a negative regulator (76, 83). Increased GRβ expression has been reported in PBMCs in response to IL-17 exposure. This subsequently hindered dexamethasone-mediated prevention of cell proliferation and apoptosis, resulting in prolonged inflammation (84). mRNA expression profiles of PBMCs (mitogen-induced kinase phosphatase 1, IL-8, and GR-β) are considered relevant parameters to predict the response of clinical asthmatics to CS (85). Indeed, PBMCs isolated from ICS-unresponsive asthmatic patients secreted significantly higher levels of IL-17A when compared to those from steroid-responsive asthmatics, and IL-17A production was inversely correlated with the clinical response to prednisolone (86). Moreover, patients with a higher IL-17 gene signature exhibited a lower response to ICS therapy in FEV_1_%-predicted-over-30-months change (8).

Recent findings by Ouyang et al. suggest that colony stimulating factor 3 (CSF3), a key neutrophil survival cytokine, mediates an IL-17A/ICS synergistic interaction leading to increased airway neutrophilic inflammation. Thus, it was demonstrated that IL-17A and dexamethasone synergistically induced *CSF3* gene expression in human ASM cells *in vitro* by increasing gene promoter activity and prevent *CSF3* mRNA degradation (87). Targeting with anti-IL-17A or the small molecule IL-17 blocker cyanidin-3-glucoside (C3G) inhibited neutrophil influx into the airways in a steroid-insensitive neutrophil/Th17-high mouse model of acute asthma, underlining the significance of CSF3 in IL-17A-mediated reduction in ICS sensitivity (87).

## Targeting the Interleukin-17 Pathway in Asthma

**Figure 2:**
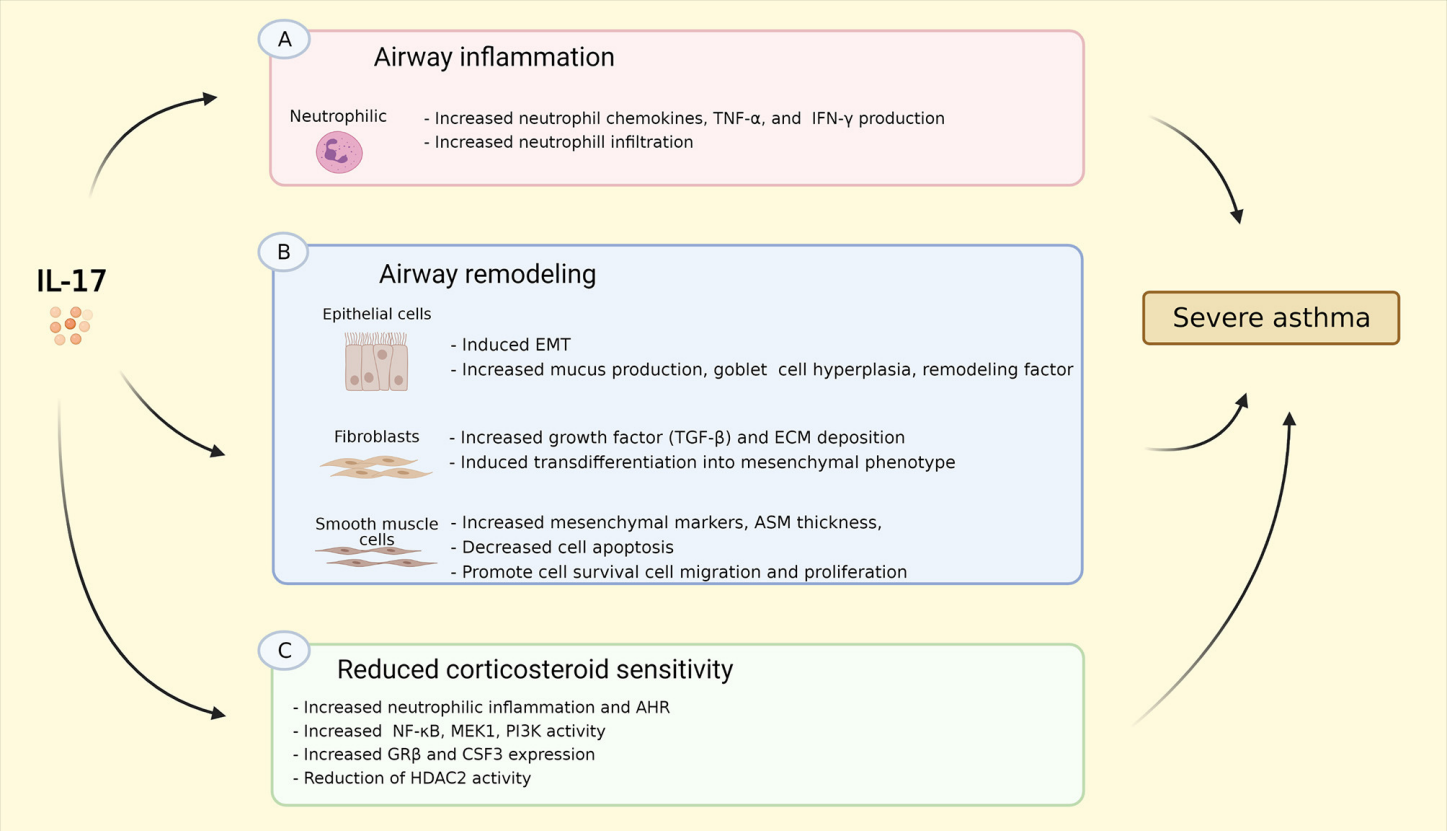
Summary of IL-17 effects on asthma. **(A)** IL-17 induced neutrophil chemokins production, e.g. CXCL1, CXCL2, CXCL5, GM-CSF, G-CSF, as well as TNF-α, and IFN-γ resulting in increased neutrophil infiltration into the airway; **(B)** IL-17 induces EMT, increases goblet cell hyperplasia, mucus, and remodeling factor production in epithelial cells; IL-17 stimulates production of TGF-β, ECM deposition as well as transdifferentiation into mesenchymal phenotype in fibroblasts; IL-17 increases mesenchymal cell markers, promotes cell survival, migration, and proliferation, and reduces cell apoptosis, resulting in increased ASM thickness. **(C)** IL-17 triggers neutrophilic inflammation and AHR *in vivo* via increasing NF-κB, MEK1, PI3K activity, GR-β and CSF3/G-CSF expression, as well as reduction of HDAC2 activity. These effects might explain the linked between Th17-high inflammation and severe asthma. IL-17, interleukin 17; CXCL-, C-X-C ligand-, GM-CSF, granulocyte-macrophage colony-stimulating factor; G-CSF, granulocyte-colony stimulating factor; TNF-α, tumor necrosis factor α; IFN-γ, interferon γ; EMT, epithelial to mesenchymal transition; TGF-β, transforming growth factor β; ECM, extracellular matrix; NF-κB, nuclear factor kappa-light-chain-enhancer of activated B cells; MEK1, MAP (Mitogen-Activated Protein) Kinase/ERK (Extracellular Signal-Regulated Kinase) Kinase 1; PI3K, phosphoinositide 3-kinase; GR-β, glucocorticoid receptor β; CSF3, colony stimulating factor 3; HDAC2, histone deacetylase 2. Created with BioRender.com.

The prevalence of heterogeneous phenotypes in severe asthma has become increasingly more evident, which makes cytokine-targeted therapies likely to be only beneficial in specific patient populations (88). The effects of IL-17 on asthma are summarized in Figure 2. In this review, we presented that IL-17 is upregulated in asthma, notably in severe asthma. Several preclinical and clinical studies reported relatively high level of IL-17, particularly IL-17A and IL-17F, in sputum, nasal and bronchial biopsies, and blood of patients with severe asthma (10, 11, 13–15, 18). Therefore, targeting IL-17/Th17 pathway may be a promising strategy, given its putative role in relevant processes in asthma, e.g. inflammation, airway remodeling, and AHR as has been discussed in this review. However, the current treatment for severe asthma is focused predominantly on targeting Th2/eosinophilic inflammation, with sputum eosinophil counts and exhaled nitric oxide fraction (FENO) as a therapy guide, and therapies primarily targeting Th2 inflammation (e.g., IL-5, anti-IgE, anti-IL-4/IL-13) as treatment options (17). Furthermore, over the last decade, approximately 78% of all randomized control trials (RCTs) on biologics under consideration for severe asthma have been targeted toward a Th2-high endotype (88).

Despite promising preclinical results that have been discussed in this review, clinical trials on IL-17 inhibition failed to demonstrate a sufficiently effective clinical response thus far. Brodalumab, a human anti-IL-17 receptor monoclonal antibody, did not meet its primary efficacy outcome [the asthma control questionnaire (ACQ)] in moderate to severe asthmatics treated with ICS (89). A significant improvement in ACQ was only observed in the high-reversibility patient subgroup (post-bronchodilator FEV1 improvement ≥20%; *n* = 112) (89). The study was well designed by anticipating the heterogeneity of the severe asthma population. Prespecified subgroup analyses were done based on nine different subgroups, based on bronchodilator reversibility, baseline FEV1% predicted, ACQ, ICS dose, FeNO level, peripheral eosinophils, sex, race, and weight (89). It should be noted that Th17-high inflammation (in which high anti-IL17 (Brodalumab) effects are expected) was not assessed in these patients. Moreover, the study populations were highly atopic (83% in total population; 79 and 84% in placebo and Brodalumab treatment arms, respectively). It has been reported that atopic asthma is more likely to correlate to an eosinophilic phenotype, therefore the subjects included in the study might not be the best target group for Brodalumab (90, 91). Interleukin−17-targeted therapy such as Brodalumab may be more beneficial in a specific Th17-high asthma subjects. Several endotyping and phenotyping strategies can be applied to define patients with Th17-high asthma, namely TAC3 gene signatures, 6GS, and IL-17 gene signatures alongside airway inflammation phenotyping (eosinophilic, mixed, neutrophilic, and paucigranulocytic) as described previously in this review (6, 8, 35, 38). Moreover, Brodalumab is proven effective in other IL-17-driven inflammatory diseases, i.e psoriasis, rheumatoid arthritis, and psoriatic arthritis, indicating its potential in Th17-high asthma (92, 93).

Phenotype-targeted clinical trials have been proven beneficial in evaluating biologic efficacies in asthma, but have not always been straightforward. Early anti-IL-5 monoclonal antibody (Mepolizumab) studies in asthmatics did not show significant results in asthma clinical measures (asthma exacerbations, AHR, FEV1, peak flow recordings) (94–96). Nevertheless, the levels of the airway and peripheral eosinophil were greatly decreased with Mepolizumab (95, 96). Only after the focus was shifted toward patients with eosinophilic asthma, the desired clinical effects of Mepolizumab on exacerbation frequency were found (97, 98). Similar lessons to focus on the phenotype-specific subjects were learned from the development of other Th-2-targeted asthma therapy, such as anti-IL-4R (Dupilumab) monoclonal antibody (mAb) (99, 100). This advocates for the evaluation of IL17-targeted therapy in the Th17 high population. In addition, considering the significance of IL-17 in reduced ICS sensitivity, it will be important to also evaluate (oral) corticosteroid use and responsiveness as endpoints.

Another potential strategy in improving IL-17-targeted therapy in asthma is by antagonizing IL-17/ IL-17 receptor interaction with small molecules instead of mABs such as Brodalumab. The limitations of mAbs are their high cost, lack of oral bioavailability, and relatively large structures, which make them difficult to be formulated as local delivery medications (i.e inhalers) (101, 102). Small molecule formulations instead of mABs might circumvent some of these problems. Indeed, small molecules have the potential to be developed into orally bioavailable drugs (103). A combined computational and hydrogen/deuterium exchange mass spectrometry (HDX-MS) study revealed an inhibitory small molecule binding site on IL-17A ligand called β-hairpin, which disrupted the interaction to its receptor (103). Recently, two small molecules (CBG040591 and CBG060392) targeting the interaction of IL-17A/IL-17RA were identified (101). Both molecules were biologically functional by preventing the production of IL-17A-induced CCL20 and CXCL-8 in human keratinocytes. Moreover, molecule CBG060392 showed partial inhibition of IL-17A intracellular signaling, suggesting a functional downstream effect (101). In another computational study, Cyanidin, a natural small molecule compound, inhibited the IL-17A/IL-17RA interaction by docking into the IL-17RA pocket (104). Further, Cyanidin was shown to attenuated skin hyperplasia in murine psoriasis model, reduced inflammation in Th17-driven murine multiple sclerosis model, and alleviated AHR in murine obesity-induced and allergic asthma model (104). These findings encourage for exploration of other potential small molecules targeting IL-17/IL-17R.

Over the last 5 years, several preclinical studies revealed (novel) key factors/mechanisms involved in Th17/IL-17-driven airway inflammation and its effects on ICS responsiveness, which markedly improved our understanding of this complex pathway and will allow for more effective therapeutic targeting. Targeted interventions in humans so far have not been rewarding, but several innovative avenues for interventions are being explored. Also, in light of the high cost of research and developing therapies, it is important to identify which patients will most likely benefit from an approach specifically aimed at Th17/IL-17 inhibition, i.e., those patients presenting with a high IL-17 gene signature and Th2-low airway inflammation. Future research is also warranted to further explore the mechanisms underpinning the effects of the Th17/IL-17 pathway on ICS responsiveness to gain better insight on how to reverse reduced ICS sensitivity and ameliorate disease severity.

## Author Contributions

SR, MV, and RG conceived and designed the draft. SR and MV wrote the draft. HK, AD, MG, and RG revised the draft. All authors contributed to the article and approved the submitted version.

## Conflict of Interest

The authors declare that the research was conducted in the absence of any commercial or financial relationships that could be construed as a potential conflict of interest.

## Publisher's Note

All claims expressed in this article are solely those of the authors and do not necessarily represent those of their affiliated organizations, or those of the publisher, the editors and the reviewers. Any product that may be evaluated in this article, or claim that may be made by its manufacturer, is not guaranteed or endorsed by the publisher.

## References

[1] Global Initiative for Asthma. Global Strategy for Asthma Management and Prevention Updated 2020. Global Initiative for Asthma. (2020). p. 20, 48, 94–109.

[2] Chung KF Wenzel SE Brozek JL Bush A Castro M Sterk PJ. International ERS/ATS guidelines on definition, evaluation and treatment of severe asthma. Eur Respir J. (2014) 43:343–73. 10.1183/09031936.00202013 24337046

[3] Israel E Reddel HK. Severe and difficult-to-treat asthma in adults. N Engl J Med. (2017) 377:965–76. 10.1056/NEJMra1608969 28877019

[4] Lambrecht BN Hammad H Fahy JV. The cytokines of asthma. Immunity. (2019) 50:975–91. 10.1016/j.immuni.2019.03.018 30995510

[5] Simpson JL Scott R Boyle MJ Gibson PG. Inflammatory subtypes in asthma: assessment and identification using induced sputum. Respirology. (2006) 11:54–61. 10.1111/j.1440-1843.2006.00784.x 16423202

[6] Chung KF. Asthma phenotyping: A necessity for improved therapeutic precision and new targeted therapies. J Intern Med. (2016) 279:192–204. 10.1111/joim.12382 26076339

[7] Choy DF Hart KM Borthwick LA Shikotra A Nagarkar DR Siddiqui S. TH2 and TH17 inflammatory pathways are reciprocally regulated in asthma. Sci Transl Med. (2015) 7:301ra129. 10.1126/scitranslmed.aab3142 26290411

[8] Christenson SA Van Den Berge M Faiz A Inkamp K Bhakta N Bonser LR. An airway epithelial IL-17A response signature identifies a steroid-unresponsive COPD patient subgroup. J Clin Invest. (2019) 129:169–81. 10.1172/JCI121087 30383540 PMC6307967

[9] Molet S Hamid Q Davoine F Nutku E Taha R Pagé N. IL-17 is increased in asthmatic airways and induces human bronchial fibroblasts to produce cytokines. J Allergy Clin Immunol. (2001) 108:430–8. 10.1067/mai.2001.117929 11544464

[10] Chakir J Shannon J Molet S Fukakusa M Elias J Laviolette M. Airway remodeling-associated mediators in moderate to severe asthma: effect of steroids on TGF-β, IL-11, IL-17, and type I and type III collagen expression. J Allergy Clin Immunol. (2003) 111:1293–8. 10.1067/mai.2003.1557 12789232

[11] Bullens DMA Truyen E Coteur L Dilissen E Hellings PW Dupont LJ. IL-17 mRNA in sputum of asthmatic patients: linking T cell driven inflammation and granulocytic influx? Respir Res. (2006) 7:135. 10.1186/1465-9921-7-135 17083726 PMC1636037

[12] Zheng R Wang F Huang Y Xiang Q Dai H Zhang W. Elevated Th17 cell frequencies and Th17/Treg ratio are associated with airway hyperresponsiveness in asthmatic children. J Asthma. (2021) 58:707–16. 10.1080/02770903.2020.1737710 32114839

[13] Doe C Bafadhel M Siddiqui S Desai D Mistry V Rugman P. Expression of the T helper 17-associated cytokines IL-17A and IL-17F in asthma and COPD. Chest. (2010) 138:1140–7. 10.1378/chest.09-3058 20538817 PMC2972626

[14] Agache I Ciobanu C Agache C Anghel M. Increased serum IL-17 is an independent risk factor for severe asthma. Respir Med. (2010) 104:1131–7. 10.1016/j.rmed.2010.02.018 20338742

[15] Ricciardolo FLM Sorbello V Folino A Gallo F Massaglia GM Favatà G. Identification of IL-17F/frequent exacerbator endotype in asthma. J Allergy Clin Immunol. (2017) 140:395–406. 10.1016/j.jaci.2016.10.034 27931975

[16] Bullone M Carriero V Bertolini F Folino A Mannelli A Di Stefano A. Elevated serum IgE, oral corticosteroid dependence and IL-17/22 expression in highly neutrophilic asthma. Eur Respir J. (2019) 54:1900068. 10.1183/13993003.00068-2019 31439682

[17] Holguin F Cardet JC Chung KF Diver S Ferreira DS Fitzpatrick A. Management of severe asthma: a european respiratory society/american thoracic society guideline. Eur Respir J. (2020) 55:1900588. 10.1183/13993003.00588-2019 31558662

[18] Al-Ramli W Préfontaine D Chouiali F Martin JG Olivenstein R Lemière C. TH17-associated cytokines (IL-17A and IL-17F) in severe asthma. J Allergy Clin Immunol. (2009) 123:1185–7. 10.1016/j.jaci.2009.02.024 19361847

[19] Liu H Mi S Li Z Hua F Hu ZW. Interleukin 17A inhibits autophagy through activation of PIK3CA to interrupt the GSK3B-mediated degradation of BCL2 in lung epithelial cells. Autophagy. (2013) 9:730–42. 10.4161/auto.24039 23514933 PMC3669182

[20] Yao Z Painter SL Fanslow WC Ulrich D Macduff BM Spriggs MK. Human IL-17: a novel cytokine derived from T cells. J Immunol. (1995) 155:5483–6. 7499828

[21] Gaffen SL. Structure and signalling in the IL-17 receptor family. Nat Rev Immunol. (2009) 9:556–67. 10.1038/nri2586 19575028 PMC2821718

[22] Swaidani S Bulek K Kang Z Liu C Lu Y Yin W. The critical role of epithelial-derived Act1 in IL-17- and IL-25-mediated pulmonary inflammation. J Immunol. (2009) 182:1631–40. 10.4049/jimmunol.182.3.1631 19155512 PMC3015148

[23] Guerra ES Lee CK Specht CA Yadav B Huang H Akalin A. Central role of IL-23 and IL-17 producing eosinophils as immunomodulatory effector cells in acute pulmonary aspergillosis and allergic asthma. PLoS Pathog. (2017) 13:e1006175. 10.1371/journal.ppat.1006175 28095479 PMC5271415

[24] Ouyang W Kolls JK Zheng Y. The biological functions of T helper 17 cell effector cytokines in inflammation. Immunity. (2008) 28:454–67. 10.1016/j.immuni.2008.03.004 18400188 PMC3424508

[25] Liu D Tan Y Bajinka O Wang L Tang Z. Th17/IL-17 axis regulated by airway microbes get involved in the development of asthma. Curr Allergy Asthma Rep. (2020) 20:11. 10.1007/s11882-020-00903-x 32172346

[26] Wilson NJ Boniface K Chan JR McKenzie BS Blumenschein WM Mattson JD. Development, cytokine profile and function of human interleukin 17-producing helper T cells. Nat Immunol. (2007) 8:950–7. 10.1038/ni1497 17676044

[27] Cho M-L Kang J-W Moon Y-M Nam H-J Jhun J-Y Heo S-B. STAT3 and NF-κB Signal pathway is required for IL-23-mediated IL-17 production in spontaneous arthritis animal model IL-1 receptor antagonist-deficient mice. J Immunol. (2006) 176:5652–61. 10.4049/jimmunol.176.9.5652 16622035

[28] Cua DJ Tato CM. Innate IL-17-producing cells: the sentinels of the immune system. Nat Rev Immunol. (2010) 10:479–89. 10.1038/nri2800 20559326

[29] Taylor PR Roy S Leal SM Sun Y Howell SJ Cobb BA. Autocrine IL-17A-IL-17RC neutrophil activation in fungal infections is regulated by IL-6, IL-23, RORγt and Dectin-2 HHS Public Access. Nat Immunol. (2014) 15:143–51. 10.1038/ni.2797 24362892 PMC3972892

[30] Moseley TA Haudenschild DR Rose L Reddi AH. Interleukin-17 family and IL-17 receptors. Cytokine Growth Factor Rev. (2003) 14:155–74. 10.1016/S1359-6101(03)00002-9 12651226

[31] Silva WA. The profile of gene expression of human marrow mesenchymal stem cells. Stem Cells. (2003) 21:661–9. 10.1634/stemcells.21-6-661 14595126

[32] Li X Bechara R Zhao J McGeachy MJ Gaffen SL. IL-17 receptor–based signaling and implications for disease. Nat Immunol. (2019) 20:1594–602. 10.1038/s41590-019-0514-y 31745337 PMC6943935

[33] Willis CR Siegel L Leith A Mohn D Escobar S Wannberg S. IL-17RA signaling in airway inflammation and bronchial hyperreactivity in allergic asthma. Am J Respir Cell Mol Biol. (2015) 53:810–21. 10.1165/rcmb.2015-0038OC 25919006

[34] Fahy JV. Type 2 inflammation in asthma-present in most, absent in many. Nat Rev Immunol. (2015) 15:57–65. 10.1038/nri3786 25534623 PMC4390063

[35] Kuo CHS Pavlidis S Loza M Baribaud F Rowe A Pandis I. T-helper cell type 2 (Th2) and non-Th2 molecular phenotypes of asthma using sputum transcriptomics in U-BIOPRED. Eur Respir J. (2017) 49:1602135. 10.1183/13993003.02135-2016 28179442

[36] Diamant Z Vijverberg S Alving K Bakirtas A Bjermer L Custovic A. Toward clinically applicable biomarkers for asthma: an EAACI position paper. Allergy Eur J Allergy Clin Immunol. (2019) 74:1835–51. 10.1111/all.13806 30953574

[37] Woodruff PG Boushey HA Dolganov GM Barker CS Yee HY Donnelly S. Genome-wide profiling identifies epithelial cell genes associated with asthma and with treatment response to corticosteroids. Proc Natl Acad Sci U S A. (2007) 104:15858–63. 10.1073/pnas.0707413104 17898169 PMC2000427

[38] Fricker M Gibson PG Powell H Simpson JL Yang IA Upham JW. A sputum 6-gene signature predicts future exacerbations of poorly controlled asthma. J Allergy Clin Immunol. (2019) 144:51–60.e11. 10.1016/j.jaci.2018.12.1020 30682452

[39] McKinley L Alcorn JF Peterson A DuPont RB Kapadia S Logar A. T H 17 cells mediate steroid-resistant airway inflammation and airway hyperresponsiveness in mice. J Immunol. (2008) 181:4089–97. 10.4049/jimmunol.181.6.4089 18768865 PMC3638757

[40] Lajoie S Lewkowich IP Suzuki Y Clark JR Sproles AA Dienger K. Complement-mediated regulation of the IL-17A axis is a central genetic determinant of the severity of experimental allergic asthma. Nat Immunol. (2010) 11:928–35. 10.1038/ni.1926 20802484 PMC2943538

[41] Newcomb DC Boswell MG Zhou W Huckabee MM Goleniewska K Sevin CM. Human TH17 cells express a functional IL-13 receptor and IL-13 attenuates IL-17A production. J Allergy Clin Immunol. (2011) 127:1006–13. 10.1016/j.jaci.2010.11.043 21236478 PMC3916096

[42] Yamanaka K Fujisawa T Kusagaya H Mori K Niwa M Furuhashi K. IL-13 regulates IL-17C expression by suppressing NF-κB-mediated transcriptional activation in airway epithelial cells. Biochem Biophys Res Commun. (2018) 495:1534–40. 10.1016/j.bbrc.2017.11.207 29203240

[43] Schnyder-Candrian S Togbe D Couillin I Mercier I Brombacher F Quesniaux V. Interleukin-17 is a negative regulator of established allergic asthma. J Exp Med. (2006) 203:2715–25. 10.1084/jem.20061401 17101734 PMC2118159

[44] Östling J van Geest M Schofield JPR Jevnikar Z Wilson S Ward J. IL-17–high asthma with features of a psoriasis immunophenotype. J Allergy Clin Immunol. (2019) 144:1198–213. 10.1016/j.jaci.2019.03.027 30998987

[45] Kim D McAlees JW Bischoff LJ Kaur D Houshel LK Gray J. Combined administration of anti-IL-13 and anti-IL-17A at individually sub-therapeutic doses limits asthma-like symptoms in a mouse model of Th2/Th17 high asthma. Clin Exp Allergy. (2019) 49:317–30. 10.1111/cea.13301 30353972 PMC6393183

[46] Pezzulo AA Tudas RA Stewart CG Vargas Buonfiglio LG Lindsay BD Taft PJ. HSP90 inhibitor geldanamycin reverts IL-13–and IL-17–induced airway goblet cell metaplasia. J Clin Invest. (2019) 129:744–58. 10.1172/JCI123524 30640172 PMC6355221

[47] Schopf FH Biebl MM Buchner J. The HSP90 chaperone machinery. Nat Rev Mol Cell Biol. (2017) 18:345–60. 10.1038/nrm.2017.20 28429788

[48] Harb H Stephen-Victor E Crestani E Benamar M Massoud A Cui Y. A regulatory T cell Notch4–GDF15 axis licenses tissue inflammation in asthma. Nat Immunol. (2020) 21:1359–70. 10.1038/s41590-020-0777-3 32929274 PMC7578174

[49] Panettieri RA. The role of neutrophils in asthma. Immunol Allergy Clin North Am. (2018) 38:629–38. 10.1016/j.iac.2018.06.005 30342584

[50] Moore WC Hastie AT Li X Li H Busse WW Jarjour NN. Sputum neutrophil counts are associated with more severe asthma phenotypes using cluster analysis. J Allergy Clin Immunol. (2014) 133:1557–63. 10.1016/j.jaci.2013.10.011 24332216 PMC4040309

[51] Backman H Jansson S-A Stridsman C Eriksson B Hedman L Eklund B-M. Severe asthma-A population study perspective. Clin Exp Allergy. (2019) 49:819–28. 10.1111/cea.13378 30817038

[52] Pelletier M Maggi L Micheletti A Lazzeri E Tamassia N Costantini C. Evidence for a cross-talk between human neutrophils and Th17 cells. Blood. (2010) 115:335–43. 10.1182/blood-2009-04-216085 19890092

[53] Essilfie AT Simpson JL Dunkley ML Morgan LC Oliver BG Gibson PG. Combined Haemophilus influenzae respiratory infection and allergic airways disease drives chronic infection and features of neutrophilic asthma. Thorax. (2012) 67:588–99. 10.1136/thoraxjnl-2011-200160 22387445

[54] Yang X Wang Y Zhao S Wang R Wang C. Long-term exposure to low-dose Haemophilus influenzae during allergic airway disease drives a steroid-resistant neutrophilic inflammation and promotes airway remodeling. Oncotarget. (2018) 9:24898–913. 10.18632/oncotarget.24653 29861841 PMC5982741

[55] Taylor SL Leong LEX Choo JM Wesselingh S Yang IA Upham JW. Inflammatory phenotypes in patients with severe asthma are associated with distinct airway microbiology. J Allergy Clin Immunol. (2018) 141:94–103.e15. 10.1016/j.jaci.2017.03.044 28479329

[56] Radaeva S Sun R Pan HN Hong F Gao B. Interleukin 22 (IL-22) plays a protective role in T cell-mediated murine hepatitis: IL-22 is a survival factor for hepatocytes via STAT3 activation. Hepatology. (2004) 39:1332–42. 10.1002/hep.20184 15122762

[57] Itoigawa Y Harada N Harada S Katsura Y Makino F Ito J. TWEAK enhances TGF-β-induced epithelial-mesenchymal transition in human bronchial epithelial cells. Respir Res. (2015) 16:48. 10.1186/s12931-015-0207-5 25890309 PMC4397832

[58] Ma L Jiang M Zhao X Sun J Pan Q Chu S. Cigarette and IL-17A synergistically induce bronchial epithelial-mesenchymal transition via activating IL-17R/NF-κB signaling. BMC Pulm Med. (2020) 20:26. 10.1186/s12890-020-1057-6 32000730 PMC6993491

[59] Evasovic JM Singer CA. Regulation of IL-17A and implications for TGF-β1 comodulation of airway smooth muscle remodeling in severe asthma. Am J Physiol - Lung Cell Mol Physiol. (2019) 316:L843–68. 10.1152/ajplung.00416.2018 30810068 PMC6589583

[60] Dessalle K Narayanan V Kyoh S Mogas A Halayko AJ Nair P. Human bronchial and parenchymal fibroblasts display differences in basal inflammatory phenotype and response to IL-17A. Clin Exp Allergy. (2016) 46:945–56. 10.1111/cea.12744 27079765

[61] Loza MJ Djukanovic R Chung KF Horowitz D Ma K Branigan P. Validated and longitudinally stable asthma phenotypes based on cluster analysis of the ADEPT study. Respir Res. (2016) 17:165. 10.1186/s12931-016-0482-9 27978840 PMC5159977

[62] Manni ML Mandalapu S McHugh KJ Elloso MM Dudas PL Alcorn JF. Molecular mechanisms of airway hyperresponsiveness in a murine model of steroid-resistant airway inflammation. J Immunol. (2016) 196:963–77. 10.4049/jimmunol.1501531 26729801 PMC4724491

[63] An SS Bai TR Bates JHT Black JL Brown RH Brusasco V. Airway smooth muscle dynamics: a common pathway of airway obstruction in asthma. Eur Respir J. (2007) 29:834–60. 10.1183/09031936.00112606 17470619 PMC2527453

[64] James AL Wenzel S. Clinical relevance of airway remodelling in airway diseases. Eur Respir J. (2007) 30:134–55. 10.1183/09031936.00146905 17601971

[65] Jeffery PK. Remodeling in asthma and chronic obstructive lung disease. Am J Respir Crit Care Med. (2001) 164:S28–38. 10.1164/ajrccm.164.supplement_2.2106061 11734464

[66] Paré PD. The functional consequences of airway remodelling in asthma. Monaldi Arch Chest Dis. (1997) 52:589–96. 9550873

[67] Kalluri R Neilson EG. Epithelial-mesenchymal transition and its implications for fibrosis. J Clin Invest. (2003) 112:1776–84. 10.1172/JCI200320530 14679171 PMC297008

[68] Hall SL Baker T Lajoie S Richgels PK Yang Y McAlees JW. IL-17A enhances IL-13 activity by enhancing IL-13–induced signal transducer and activator of transcription 6 activation. J Allergy Clin Immunol. (2017) 139:462–71.e14. 10.1016/j.jaci.2016.04.037 27417023 PMC5149451

[69] Peters M Köhler-Bachmann S Lenz-Habijan T Bufe A. Influence of an allergen-specific Th17 response on remodeling of the airways. Am J Respir Cell Mol Biol. (2016) 54:350–8. 10.1165/rcmb.2014-0429OC 26222011

[70] Fujisawa T Velichko S Thai P Hung L-Y Huang F Wu R. Regulation of airway MUC5AC expression by IL-1β and IL-17A; the NF-κB paradigm. J Immunol. (2009) 183:6236–43. 10.4049/jimmunol.0900614 19841186 PMC4623590

[71] Fogli LK Sundrud MS Goel S Bajwa S Jensen K Derudder E. T Cell–derived IL-17 mediates epithelial changes in the airway and drives pulmonary neutrophilia. J Immunol. (2013) 191:3100–11. 10.4049/jimmunol.1301360 23966625 PMC3822005

[72] Lai T Tian B Cao C Hu Y Zhou J Wang Y. HDAC2 suppresses IL17A-mediated airway remodeling in human and experimental modeling of COPD. Chest. (2018) 153:863–75. 10.1016/j.chest.2017.10.031 29113816

[73] Zhang J Wang D Wang L Wang S Roden AC Zhao H. Profibrotic effect of IL-17A and elevated IL-17RA in idiopathic pulmonary fibrosis and rheumatoid arthritis-associated lung disease support a direct role for IL-17A/IL-17RA in human fibrotic interstitial lung disease. Am J Physiol - Lung Cell Mol Physiol. (2019) 316:L487–97. 10.1152/ajplung.00301.2018 30604628

[74] Chang Y Al-Alwan L Risse PA Roussel L Rousseau S Halayko AJ. TH17 cytokines induce human airway smooth muscle cell migration. J Allergy Clin Immunol. (2011) 127:1046–53.e1-2. 10.1016/j.jaci.2010.12.1117 21345484

[75] Chang Y Al-Alwan L Risse PA Halayko AJ Martin JG Baglole CJ. Th17-associated cytokines promote human airway smooth muscle cell proliferation. FASEB J. (2012) 26:5152–60. 10.1096/fj.12-208033 22898922

[76] Barnes PJ. Corticosteroid resistance in patients with asthma and chronic obstructive pulmonary disease. J Allergy Clin Immunol. (2013) 131:636–45. 10.1016/j.jaci.2012.12.1564 23360759

[77] Ito K Chung KF Adcock IM. Update on glucocorticoid action and resistance. J Allergy Clin Immunol. (2006) 117:522–43. 10.1016/j.jaci.2006.01.032 16522450

[78] Wadhwa R Dua K Kim R Hansbro P Horvat J Adcock I. Cellular mechanisms underlying steroid-resistant asthma. Eur Respir Rev. (2019) 28:190096. 10.1183/16000617.0096-2019 31636089 PMC9488801

[79] Ito K Herbert C Siegle JS Vuppusetty C Hansbro N Thomas PS. Steroid-resistant neutrophilic inflammation in a mouse model of an acute exacerbation of asthma. Am J Respir Cell Mol Biol. (2008) 39:543–50. 10.1165/rcmb.2008-0028OC 18474669 PMC2643207

[80] Irvin C Zafar I Good J Rollins D Christianson C Gorska MM. Increased frequency of dual-positive TH2/TH17 cells in bronchoalveolar lavage fluid characterizes a population of patients with severe asthma. J Allergy Clin Immunol. (2014) 134:1175–86.e7. 10.1016/j.jaci.2014.05.038 25042748 PMC4254017

[81] Zijlstra GJ Ten Hacken NHT Hoffmann RF Van Oosterhout AJM Heijink IH. Interleukin-17A induces glucocorticoid insensitivity in human bronchial epithelial cells. Eur Respir J. (2012) 39:439–45. 10.1183/09031936.00017911 21828034

[82] Vazquez-Tello A Semlali A Chakir J Martin JG Leung DY Eidelman DH. Induction of glucocorticoid receptor-β expression in epithelial cells of asthmatic airways by T-helper type 17 cytokines. Clin Exp Allergy. (2010) 40:1312–22. 10.1111/j.1365-2222.2010.03544.x 20545708

[83] Pujols L Mullol J Picado C. Alpha and beta glucocorticoid receptors: relevance in airway diseases. Curr Allergy Asthma Rep. (2007) 7:93–9. 10.1007/s11882-007-0005-3 17437678

[84] Vazquez-Tello A Halwani R Hamid Q Al-Muhsen S. Glucocorticoid receptor-beta up-regulation and steroid resistance induction by IL-17 and IL-23 cytokine stimulation in peripheral mononuclear cells. J Clin Immunol. (2013) 33:466–78. 10.1007/s10875-012-9828-3 23160983

[85] Goleva E Jackson LP Gleason M Leung DYM. Usefulness of PBMCs to predict clinical response to corticosteroids in asthmatic patients. J Allergy Clin Immunol. (2012) 129:687–93.e1. 10.1016/j.jaci.2011.12.001 22236730 PMC3294080

[86] Chambers ES Nanzer AM Pfeffer PE Richards DF Timms PM Martineau AR. Distinct endotypes of steroid-resistant asthma characterized by IL-17Ahigh and IFN-γhigh immunophenotypes: potential benefits of calcitriol. J Allergy Clin Immunol. (2015) 136:628–37.e4. 10.1016/j.jaci.2015.01.026 25772594 PMC4559139

[87] Ouyang S Liu C Xiao J Chen X Lui AC Li X. Targeting IL-17A/glucocorticoid synergy to CSF3 expression in neutrophilic airway diseases. JCI Insight. (2020) 5:e132836. 10.1172/jci.insight.132836 32051346 PMC7098787

[88] Brown T Jones T Gove K Barber C Elliott S Chauhan A. Randomised controlled trials in severe asthma: selection by phenotype or stereotype. Eur Respir J. (2018) 52:1801444. 10.1183/13993003.01444-2018 30361247

[89] Busse WW Holgate S Kerwin E Chon Y Feng JY Lin J. Randomized, double-blind, placebo-controlled study of brodalumab, a human anti-IL-17 receptor monoclonal antibody, in moderate to severe asthma. Am J Respir Crit Care Med. (2013) 188:1294–302. 10.1164/rccm.201212-2318OC 24200404

[90] Snijders D Agostini S Bertuola F Panizzolo C Baraldo S Turato G. Markers of eosinophilic and neutrophilic inflammation in bronchoalveolar lavage of asthmatic and atopic children. Allergy Eur J Allergy Clin Immunol. (2010) 65:978–85. 10.1111/j.1398-9995.2009.02282.x 20002661

[91] Bradley BL Azzawi M Jacobson M Assoufi B Collins JV Irani A Marie A. Eosinophils, T-lymphocytes, mast cells, neutrophils, and macrophages in bronchial biopsy specimens from atopic subjects with asthma: comparison with biopsy specimens from atopic subjects without asthma and normal control subjects and relationship to bronc. J Allergy Clin Immunol. (1991) 88:661–74. 10.1016/0091-6749(91)90160-P 1918731

[92] Nirula A Nilsen J Klekotka P Kricorian G Erondu N Towne JE. Effect of IL-17 receptor A blockade with brodalumab in inflammatory diseases. Rheumatol. (2016) 55:ii43–55. 10.1093/rheumatology/kew346 27856660

[93] Bauer E Lucier J Furst DE. Brodalumab-An IL-17RA monoclonal antibody for psoriasis and psoriatic arthritis. Expert Opin Biol Ther. (2015) 15:883–93. 10.1517/14712598.2015.1045410 25985813

[94] Leckie MJ Ten Brinke A Khan J Diamant Z O'xonnor BJ Walls CM. Effects of an interleukin-5 blocking monoclonal antibody on eosinophils, airway hyper-responsiveness, and the late asthmatic response. Lancet. (2000) 356:2144–8. 10.1016/S0140-6736(00)03496-6 11191542

[95] Flood-Page P Swenson C Faiferman I Matthews J Williams M Brannick L. A study to evaluate safety and efficacy of mepolizumab in patients with moderate persistent asthma. Am J Respir Crit Care Med. (2007) 176:1062–71. 10.1164/rccm.200701-085OC 17872493

[96] Flood-Page PT Menzies-Gow AN Kay AB Robinson DS. Eosinophil's role remains uncertain as anti-interleukin-5 only partially depletes numbers in asthmatic airway. Am J Respir Crit Care Med. (2003) 167:199–204. 10.1164/rccm.200208-789OC 12406833

[97] Nair P Pizzichini MMM Kjarsgaard M Inman MD Efthimiadis A Pizzichini E. Mepolizumab for prednisone-dependent asthma with sputum eosinophilia. N Engl J Med. (2009) 360:985–93. 10.1056/NEJMoa0805435 19264687

[98] Haldar P Brightling CE Hargadon B Gupta S Monteiro W Sousa A. Mepolizumab and exacerbations of refractory eosinophilic asthma. N Engl J Med. (2009) 360:973–84. 10.1056/NEJMoa0808991 19264686 PMC3992367

[99] Agache I Song Y Rocha C Beltran J Posso M Steiner C. Efficacy and safety of treatment with dupilumab for severe asthma: a systematic review of the EAACI guidelines—Recommendations on the use of biologicals in severe asthma. Allergy Eur J Allergy Clin Immunol. (2020) 75:1058–68. 10.1111/all.14221 32154939

[100] Pavord ID Siddiqui S Papi A Corren J Sher LD Bardin P. Dupilumab efficacy in patients stratified by baseline treatment intensity and lung function. J Asthma Allergy. (2020) 13:701–11. 10.2147/JAA.S275068 33364789 PMC7751293

[101] Álvarez-Coiradas E Munteanu CR Díaz-Sáez L Pazos A Huber KVM Loza MI. Discovery of novel immunopharmacological ligands targeting the IL-17 inflammatory pathway. Int Immunopharmacol. (2020) 89:107026. 10.1016/j.intimp.2020.107026 33045560

[102] Arkin MMR Wells JA. Small-molecule inhibitors of protein-protein interactions: progressing towards the dream. Nat Rev Drug Discov. (2004) 3:301–17. 10.1038/nrd1343 15060526

[103] Espada A Broughton H Jones S Chalmers MJ Dodge JA. A binding site on IL-17A for inhibitory macrocycles revealed by hydrogen/deuterium exchange mass spectrometry. J Med Chem. (2016) 59:2255–60. 10.1021/acs.jmedchem.5b01693 26854023

[104] Liu C Zhu L Fukuda K Ouyang S Chen X Wang C. The flavonoid cyanidin blocks binding of the cytokine interleukin-17A to the IL-17RA subunit to alleviate inflammation *in vivo*. Sci Signal. (2017) 10:eaaf8823. 10.1126/scisignal.aaf8823 28223414 PMC5520994

